# Capturing Initial Understanding and Impressions of Surgical Therapy for Parkinson's Disease

**DOI:** 10.3389/fneur.2021.605959

**Published:** 2021-03-04

**Authors:** Somnath Das, Caio M. Matias, Sunidhi Ramesh, Lohit Velagapudi, Julie P. Barbera, Samantha Katz, Michael P. Baldassari, Mohammad Rasool, Daniel Kremens, Jeffrey Ratliff, Tsao-Wei Liang, Chengyuan Wu

**Affiliations:** ^1^Sidney Kimmel Medical College, Thomas Jefferson University, Philadelphia, PA, United States; ^2^Department of Neurosurgery, Thomas Jefferson University, Philadelphia, PA, United States; ^3^Department of Neurology, Thomas Jefferson University, Philadelphia, PA, United States

**Keywords:** DBS, Parkinson's disease, patient perspectives, patient education, patient-doctor relationship

## Abstract

**Background:** Deep Brain Stimulation (DBS) is an underutilized surgical therapy for Parkinson's Disease (PD). Both physician and patient hesitancies have been described as potential barriers to DBS, but the specifics of patient perceptions of DBS have not been well-characterized in the general PD population.

**Objective:** To characterize the understanding and impressions of surgical therapy in PD patients prior to formal surgical evaluation.

**Methods:** A 30-question survey assessing impressions of surgical therapy for PD and understanding of DBS for PD was administered to PD patients seen at an urban movement disorders clinic.

**Results:** One hundred and two patients completed the survey. When asked if they would undergo a hypothetical risk-free, curative brain surgery for PD, 98 patients responded “yes.” Patients were more agreeable to “reversible,” “minimally-invasive,” and “incisionless” surgery. 51.2% thought DBS is an “effective” treatment for PD, 76.6% thought it was “invasive,” and 18.3% thought it was “reversible.” 45.2% reported fear of being awake during DBS surgery. Regarding costs, 52.4% were concerned that DBS was “very expensive” or “not covered by insurance.” Initial source of information and perceived treatment effectiveness were not associated with concerns about DBS effectiveness or threats to normality. Negative perceptions of past surgery were associated with concerns about DBS altering mood and personality.

**Conclusion:** Overall, patients expressed concerns regarding procedural efficacy, invasiveness, cost, and irreversibility—independent of the original source of information. Future studies are required to allow us to better understand the impact of these initial findings on DBS hesitancy and underutilization.

## Introduction

Deep Brain Stimulation (DBS) is an effective surgical therapy for PD, showing benefit for motor complications as well as improvement in quality of life (1–6). It is a cost-effective treatment (7) with clear long-term benefit and high patient satisfaction (8–10). Despite the existing evidence, DBS therapy for PD may be underutilized (11), with current literature noting that as few as 10–15% of eligible PD patients are referred to DBS treatment centers (12). Physician hesitancy has been identified as a potential factor for DBS underutilization (12). Patient-specific barriers have also been identified; and it is estimated that approximately half of advanced PD patients undergoing surgery express reluctance prior to implantation (13). Given the salient role of patient impressions and understanding in choosing to undergo certain neurosurgical procedures (14, 15), it is possible that similar trends could be influencing DBS utilization.

Patients are often the drivers of the decision to undergo DBS, with about half of patients taking their own initiative when considering treatment (16). Despite the significant role of patient perspective, few studies have examined how patients conceptualize DBS prior to considering surgery (17). To date, no studies have examined impressions of DBS invasiveness or effectiveness in the general PD population, prior to discussing implantation with surgical providers. Since the few existing studies on patient perceptions of DBS have been restricted to those who ultimately undergo surgical evaluation, their findings may not be representative of the larger PD population.

As such, we aimed to characterize the understanding and impressions of surgical therapy in a general population of PD patients prior to formal surgical evaluation. We therefore developed this pilot investigation to provide a foundational understanding of how patients at an urban, tertiary movement disorders clinic learn about and perceive DBS therapy.

## Methods

This study was approved by the local Institutional Review Board. Informed consent was obtained following patient recruitment prior to survey administration. Data was collected and relevant personal identifiers were removed before analysis in order to protect patient privacy. We confirm that we have read the Journal's position on issues involved in ethical publication and affirm that this work is consistent with those guidelines.

### Survey Design

A survey was created in order to capture impressions of hypothetical surgical treatment of PD and of DBS therapy. The survey consisted of 30 questions (Supplementary Figure 1), designed by two medical students (SD and MB), a Movement Disorders Neurologist (TW), and a Functional Neurosurgeon (CW). Questions were developed based on prior studies (14, 17) and common patient impressions previously noticed by the clinicians. The survey also contained additional questions about past surgical history in order to account for possible missing data in the medical records.

The questions were intentionally designed to allow participants to interpret specific terms that are commonly used to describe surgical procedures. The phrasing of questions was therefore matched across series of questions while changing the term of interest. Patients were not allowed to discuss their interpretation of the questions with their provider or study administrators. The survey did not have “I do not know” or free-text options, and thus participant responses were limited by the their sole interpretation of each survey question.

### Patient Selection

Consecutive patients with a diagnosis of PD were selected to participate in the survey by a movement disorder neurologist (TL, DK, and JR) after a routine scheduled evaluation. Selection criteria included patients who had not undergone DBS implantation or referral to a neurosurgeon for consultation regarding DBS surgery. All DBS-naïve patients were eligible, and quality of their DBS candidacy was not considered. Having had prior discussions of DBS with a neurologist or other healthcare provider were not cause for exclusion. Patients with cognitive impairment that would preclude independent survey participation (e.g., dependence on a care-giver to respond to survey), as judged by their neurologist, were excluded from the study.

### Survey Administration

A research assistant was present to administer and collect the survey. Patients were required to complete the questionnaire based on their own interpretation of the questions and with the assistant available to answer questions but provide minimal direction in order to reduce bias. Following completion of the survey, the researcher interviewed patients to complete the Schwab & England Activities of Daily Living Scale (SE).

### Demographic and Clinical Data

Demographic variables including age and sex were collected. Clinical data collected from chart review included the MDS-UPDRS III score at the time of the visit, Levodopa Equivalent Daily Dose (LEDD) at time of visit, and years since diagnosis.

### Statistical Analysis

Statistical analyses were performed using RStudio 1.2.1335 (RStudio, Inc., Boston, MA). The distribution of continuous variables was tested using direct visualization of histograms and the Shapiro-Wilks test. Spearman's rho was used to evaluate the correlation between the demographic variables estimating the severity of disease and the willingness to undergo DBS. The source of DBS information was evaluated as a potential influence factor for impressions of DBS. The Mann-Whitney test was used for a binary analysis (source of information = physician vs. other sources). Likewise, the Kruskal-Wallis test, along with the Dunn's *post-hoc* test, was used to test if experience with previous surgeries could influence the perceptions of DBS. Any variable with a *p*-value < 0.05 was considered to be statistically significant.

## Results

### Demographics

A total of 104 patients completed the survey between November 2018 and February 2020 (Table 1). Two patients were excluded due to one requiring a caregiver to fill out the survey and another having had the DBS procedure prior to enrollment.

**Table 1:** Patient demographics.

**Demographics**	
*N*	102
Age	67.66 ± 9.13
Sex	39.2% F
Years since diagnosis	6.02 ± 3.61 (1–16)
Average UPDRS score	18.27 ±.67 (1–45)
Average LEDD score	601.73 ± 9.67 (0–2,910)
Average Schwab and England score	83.35 ± 17.20 (10–100)
Previous surgery	88 Yes (86.3%)
Impression of prior surgery	70.5% Positive
Familiar with DBS	82 Yes (80.4%)

*UPDRS, Unified Parkinson's Disease Rating Scale; LEDD, Levodopa Equivalent Daily Dose; DBS, Deep Brain Stimulation*.

### Regarding PD and Hypothetical Surgeries

**Figure 1:**
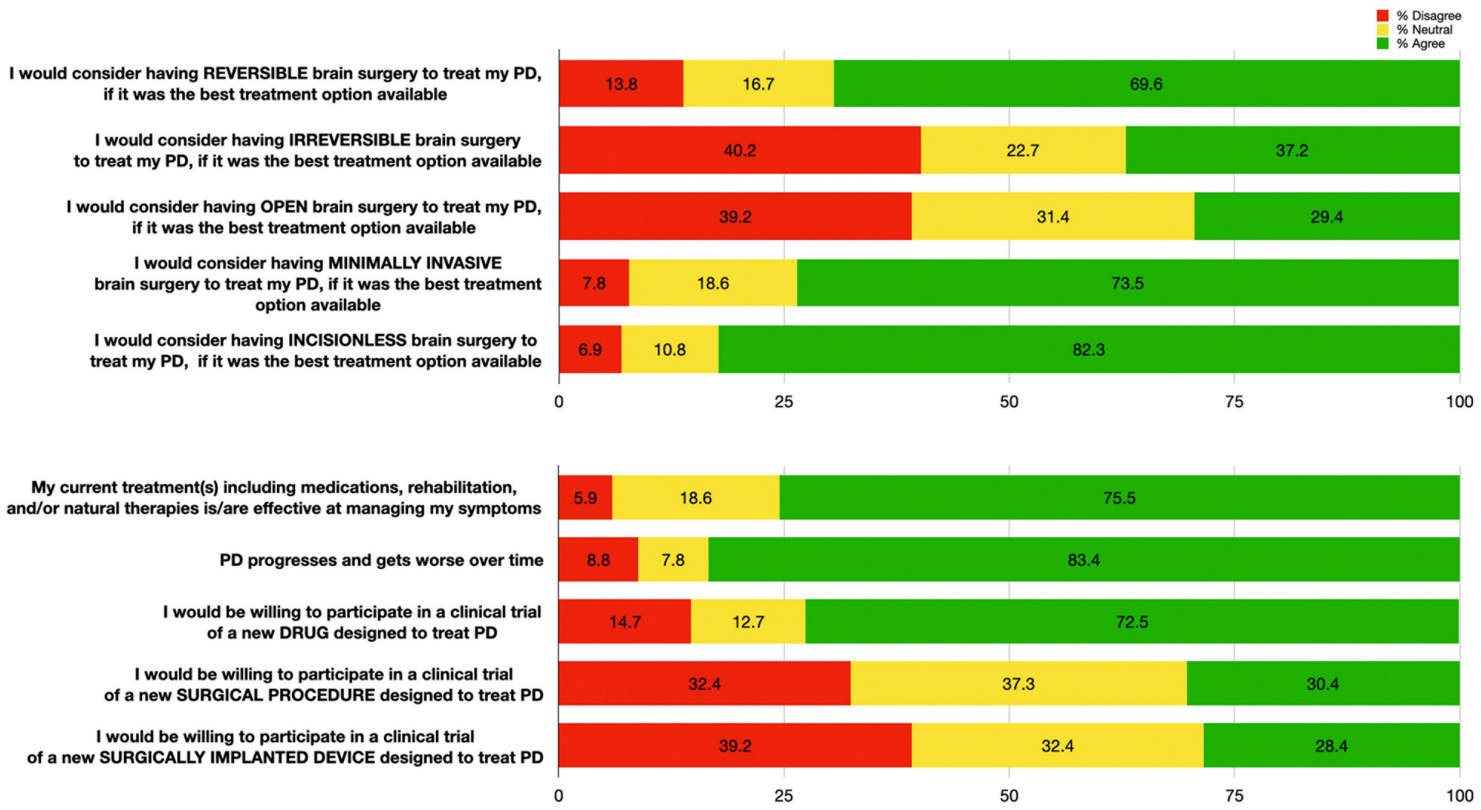
Patient responses (*n* = 102) to questions regarding impressions of hypothetical brain surgeries (top); and to questions regarding efficacy of their treatment, Parkinson's Disease progression, and openness to novel therapies (bottom). Percentages for each response category are shown: “Agree” (responses of 4 or 5, in green), “Neutral” (response of 3, in yellow), and “Disagree” (responses of 1 or 2, in red).

Most patients who reported a prior surgical history (including non-brain related surgeries) were satisfied (70.5%) with their previous procedure (Table 1). When asked if they would undergo a *hypothetical, risk-free, curative brain surgery* for their PD, 98 patients (96.1%) responded “yes.” Assuming that surgery would be the best treatment option available, most patients (>69.6%) would consider either *reversible, minimally-invasive*, or *incisionless* surgery (Figure 1). Severity of disease (as estimated by the UPDRS III, LEDD, and SE) was not correlated with the willingness to undergo DBS (Spearman's rho = 0.07, −0.05, −0.01, and *p* = 0.51, *p* = 0.68, *p* = 0.97, respectively).

Most patients agreed that PD progresses and worsens over time (83.4%), but were also satisfied with their current treatment (75.5%). Over twice as many patients reported that they would participate in PD clinical trials for drug therapies (72.5%) compared to surgical therapies (30.4%) (Figure 1).

### Impressions of DBS

**Figure 2:**
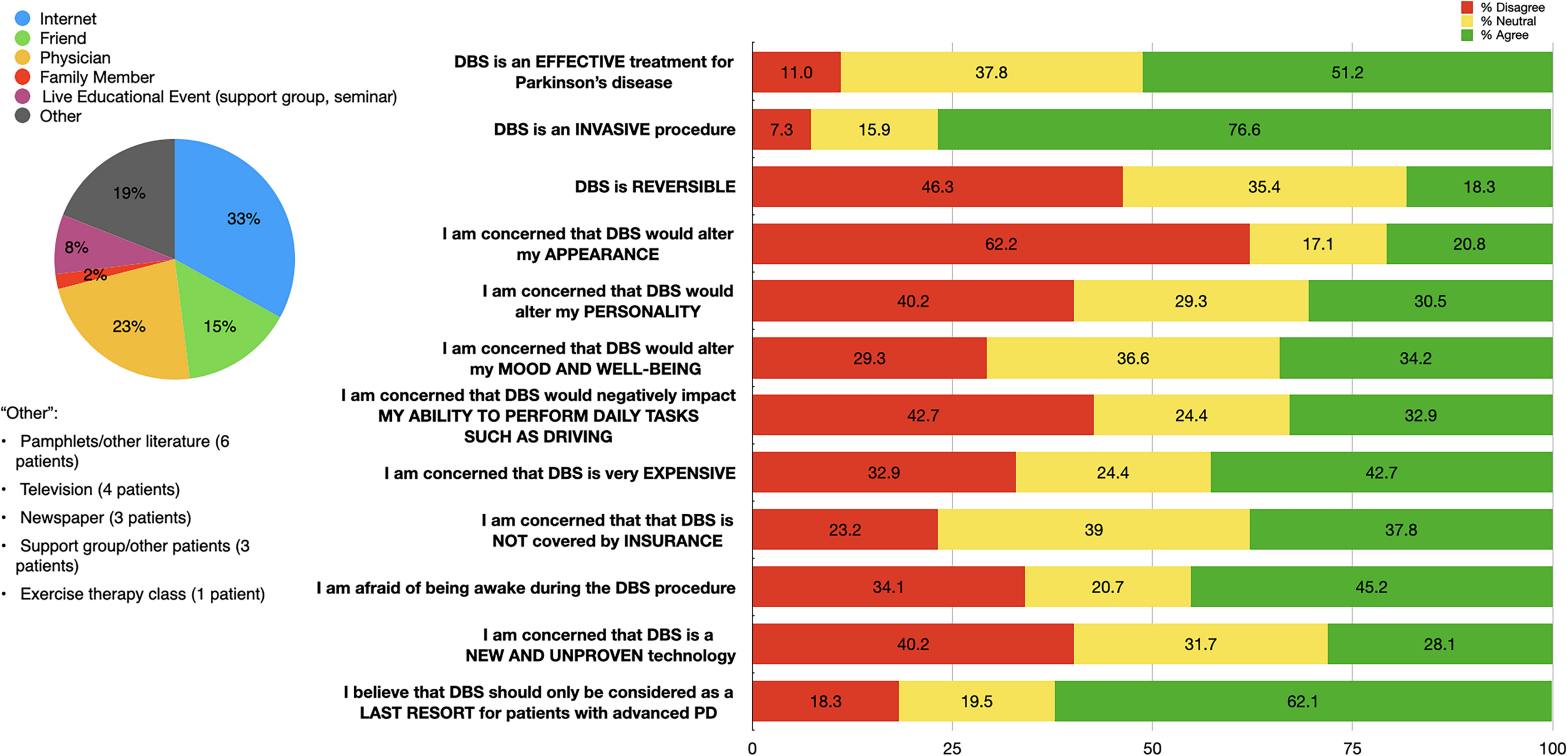
Distribution of initial sources of information (left); and patient responses to questions regarding DBS impressions (right) among our DBS-aware cohort (*n* = 82). Percentages for each response category are shown: “Agree” (responses of 4 or 5, in green), “Neutral” (response of 3, in yellow), and “Disagree” (responses of 1 or 2, in red).

Eighty-two (80.3%) patients reported familiarity with DBS, with the majority having first heard about it from the internet or a physician (Figure 2).

Approximately half (51.2%) of our cohort agreed that DBS is *effective* for PD. Most patients thought that DBS is *invasive* (76.6%) and only *as a last resort* (62.1%). A plurality of patients (46.3%) believe that DBS is not *reversible*. Smaller portions of our cohort expressed concerns about DBS altering appearance (20.8%), personality (30.5%), mood and well-being (34.2%), or negative impacts on activities of daily living (32.9%). Almost half of our cohort (45.2%) expressed fears about being awake during the DBS procedure. The majority of patients (76.8%) expressed concerns or were neutral when reporting impressions of DBS insurance coverage. Over half (52.4%) of our cohort reported concerns about either expense or insurance coverage (Figure 2).

**Figure 3:**
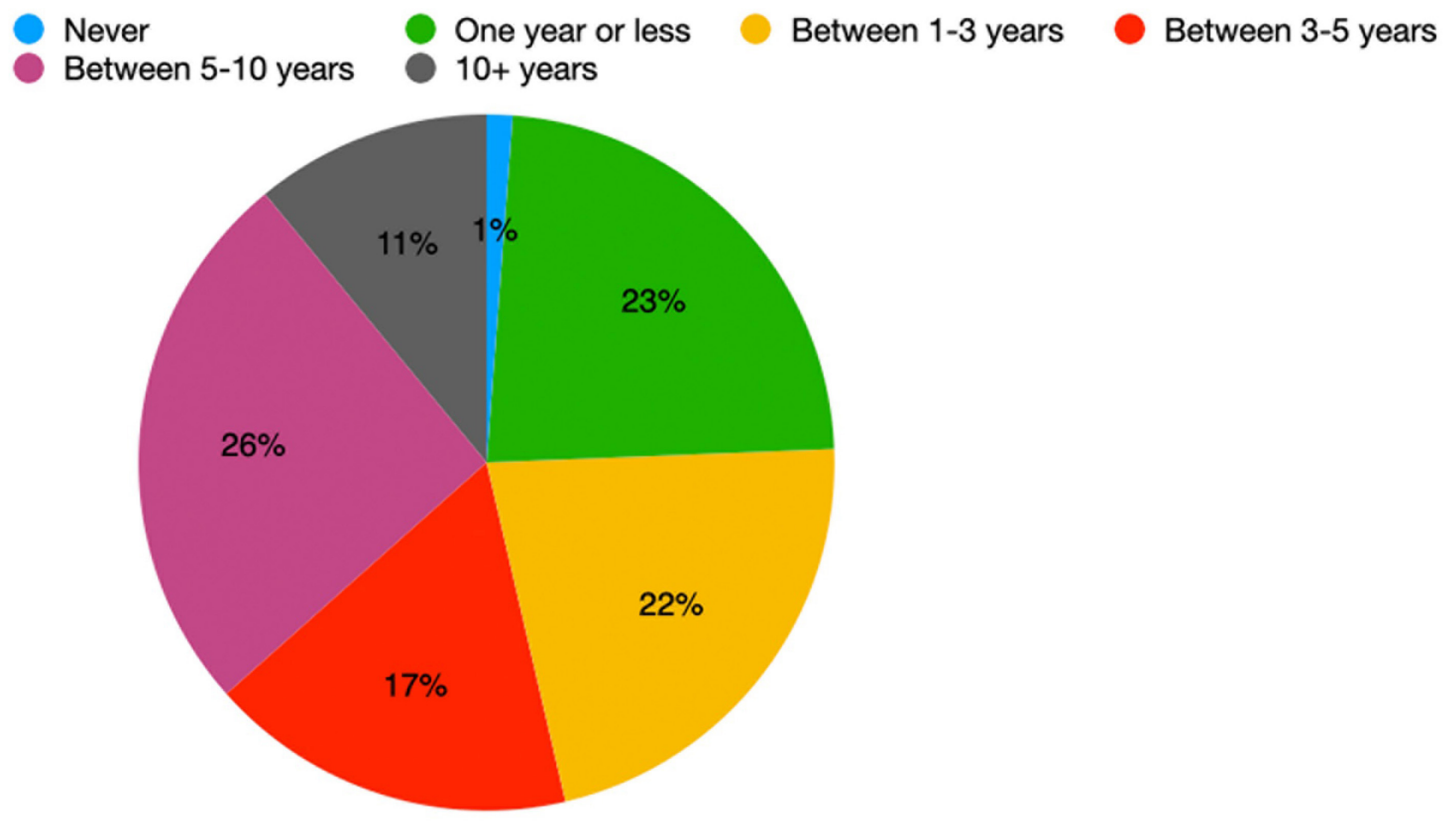
Distribution of the earliest time interval in which DBS-aware patients would consider DBS (*n* = 82).

Patients held various opinions about the time period after which they would consider undergoing DBS should their symptoms worsen. A single patient said they would *never consider DBS*; 11% of patients would *consider DBS in 10 or more years*, and 26% of patients would *consider DBS in 1 year or less*. The remaining percentage of our cohort was split almost evenly amongst patients agreeing to *DBS in 1–3 years* (22%)*, 3–5 years* (17%)*, and 5–10 years* (26%) (Figure 3).

### Factors Influencing Impressions of DBS

We analyzed if the source of DBS information could affect the impressions on DBS. Patients reported myriad sources with regards to where they became familiar with DBS (Figure 2). A physician being the source of information did not influence the perceptions of DBS (Table 2). In addition, we also evaluated if their experience of previous surgeries could affect the impressions on DBS. In those who identified prior surgery as a negative experience, we found a higher degree of concerns about DBS *altering mood and emotional well-being* (*p*-value = 0.02, *post-hoc* Dunn test: *p* = 0.05). There were no similar findings when subjects were asked about concerns regarding DBS being *effective, invasive, reversible, expensive, covered by insurance, new and unproven, a last resort procedure*, and *altering personality, appearance, or activities of daily living* (Table 3).

**Table 2:** Sources of information vs. responses to DBS impressions.

**Variable**	**Level**	**Physician (*n* = 23)**	**Other (*n* = 59)**	**Total (*n* = 82)**	***p*-value**
DBS is an effective treatment for PD	median [iqr]	4 [3, 4]	3 [3, 4]	4 [3, 4]	0.44
DBS is an invasive procedure	median [iqr]	4 [4, 5]	4 [3, 5]	4 [4, 5]	0.19
DBS is reversible	median [iqr]	2 [2, 3]	3 [2, 3]	3 [2, 3]	0.35
I am concerned that DBS is very expensive	median [iqr]	3 [2, 5]	3 [2, 4]	3 [2, 4]	0.57
I am concerned that DBS is not covered by insurance	median [iqr]	3 [2.5, 5.0]	3 [3, 4]	3 [3, 4]	0.51
I am concerned that DBS is a new and unproven technology	median [iqr]	3 [2, 4]	3 [2, 3]	3 [2, 4]	0.21
I believe that DBS should only be considered a last resort therapy for patients with advanced PD	median [iqr]	4 [3, 5]	4 [3, 5]	4 [3, 5]	0.94
I am concerned that DBS would alter my appearance	median [iqr]	2 [1, 3]	2 [1, 3]	2 [1, 3]	0.71
I am concerned that DBS would alter my personality	median [iqr]	3 [2, 4]	3 [2, 4]	3 [2, 4]	0.44
I am concerned that DBS would alter my mood	median [iqr]	3 [3, 4]	3 [2, 4]	3 [2, 4]	0.67
I am concerned that DBS would alter my ability to perform daily tasks such as driving	median [iqr]	3 [2, 4]	3 [2, 4]	3 [2, 4]	0.92

*1 = Strongly Disagree, 2 = Disagree, 3 = Neutral, 4 = Agree, 5 = Strongly Agree*.

**Table 3:** Perception of prior surgery vs. responses to DBS impressions.

**Variable**		**Negative (*n* = 5)**	**Somewhat negative (*n* = 5)**	**Neutral (*n* = 13)**	**Somewhat positive (*n* = 10)**	**Positive (*n* = 36)**	**Total (*n* = 82)**	***p*-value**
DBS is an effective treatment for PD	Median [iqr]	4 [3, 4]	4 [3, 5]	4 [3, 4]	3 [3.0, 3.8]	4 [3, 4]	4 [3, 4]	0.71
DBS is an invasive procedure	Median [iqr]	3 [3, 4]	4 [4, 5]	5 [4, 5]	4 [4, 4]	4 [3.8, 5.0]	4 [4, 5]	0.45
DBS is reversible	Median [iqr]	2 [24,444, 3]	2 [2, 3]	2 [1, 3]	3 [2.2, 3.8]	2.5 [2, 3]	3 [2, 3]	0.53
I am concerned that DBS is very expensive	Median [iqr]	4 [3, 5]	4 [3, 4]	3 [1, 4]	4 [3.2, 4.8]	3 [2, 4]	3 [2, 4]	0.39
I am concerned that DBS is not covered by insurance	Median [iqr]	3 [3, 4]	4 [3, 5]	3 [3, 4]	3.5 [3.0, 4.8]	3 [2.0, 4.2]	3 [3, 4]	0.65
I am concerned that DBS is a new and unproven technology	Median [iqr]	3 [2, 4]	3 [2, 3]	4 [3, 4]	2.5 [1, 4]	3 [2, 3]	3 [2, 4]	0.31
I believe that DBS should only be considered a last resort therapy for patients with advanced PD	Median [iqr]	4 [3, 5]	4 [3, 4]	3 [2, 5]	4 [3.2, 4.0]	4 [3, 5]	4 [3, 5]	0.89
I am concerned that DBS would alter my appearance	Median [iqr]	2 [1, 5]	3 [2, 3]	2 [1, 3]	2.5 [1.2, 4.0]	2 [1, 3]	2 [1, 3]	0.44
I am concerned that DBS would alter my personality	Median [iqr]	3 [3, 5]	3 [2, 3]	3 [1, 4]	3.5 [2.0, 4.8]	3 [1.8, 3.2]	3 [2, 4]	0.5
I am concerned that DBS would alter my mood	Median [iqr]	4 [4, 5]	3 [3, 3]	4 [3, 5]	3 [2, 4]	3 [2, 3]	3 [2, 4]	0.02*
I am concerned that DBS would alter my ability to perform daily tasks such as driving	Median [iqr]	4 [3, 5]	2 [1, 4]	3 [2, 4]	2.5 [2, 4]	3 [2, 4]	3 [2, 4]	0.43

1 = Somewhat Negative, 2 = Negative, 3 = Neutral, 4 = Somewhat Positive, 5 = Positive. For “I am concerned that DBS would alter my mood,” P = 0.05* after correction for multiple comparisons with Dunn test.

## Discussion

In this study, we surveyed 102 patients with PD who had not been formally evaluated for DBS to obtain an initial understanding about their attitude toward PD surgery and their understanding of DBS. The results of our study help to further capture patient attitudes toward surgery and DBS for treatment of their PD. Furthermore, our study's results begin to capture how patients may view commonly used descriptions of DBS, such as the procedure being “reversible” or “non-invasive.” Most notably, only approximately half of our DBS-aware patients thought that it is an *effective* treatment for PD. In addition, cost or insurance coverage was a concern for the majority of patients. Such impressions and concerns regarding DBS may contribute to patients' reluctance to even initiate discussions with a neurosurgeon—as fewer than 30% of eligible patients may agree to further evaluation at specialized DBS treatment centers (18).

### DBS Knowledge and Initial Sources of Information

Consistent with prior studies, our data demonstrates that most patients first learn about DBS either from the internet or from a physician (17, 19, 20). While it has been suggested that internet-based patient testimonials may inflate patient expectations of DBS (21), we found no correlation between patient impressions of DBS and the initial source of their information. At the same time, our survey did not allow subjects to specify the details of the internet sources. Little is known about communication regarding DBS in online forums; however, the role of interactions between previously implanted patients and those considering surgery may be an important factor in patient impressions (19). A 1-year ethnographic study of DBS internet forums revealed how implanted patients served as sources of help for patients considering the procedure (22) and that few healthcare providers participated in these forums.

In our cohort, learning about DBS from a physician was not correlated with differences in the impressions of multiple aspects of DBS surgery, suggesting that even patients who are informed about DBS by a physician may still believe it to be ineffective. This finding is particularly noteworthy as it suggests that despite physicians' efforts, patients remain uninformed about DBS efficacy. This discrepancy may suggest that patients may consider information from healthcare providers and outside sources to be equally valid, even if the latter may be inaccurate. Alternatively, patients may ultimately rely on multiple resources with conflicting information in the process of researching DBS. The effectiveness of physician communication may also be variable. It has also been reported that 2 h after participating in informed consent for a neurosurgical procedure, patients retain <20% of the information (23). Therefore, patients may refer to online resources and patient testimonials to fill in gaps of understanding. With the use of multiple resources, the initial source of information may not be particularly relevant to shaping the entire impression patients have of DBS surgery.

### Impressions of DBS Efficacy

Approximately half of our DBS-aware cohort agreed that it is an *effective* treatment for PD; yet almost 40% of patients reported that they were uncertain about DBS efficacy. This represents a disconnect between the impression of DBS efficacy and the existing evidence for DBS (1–10). This finding is especially salient given the patient-driven nature of the decision to undergo DBS reported in previous studies (16, 24). While the reasons for this misinformation among patients is not clearly evident in our data, we believe that it raises sufficient concern to justify further investigation of the population characterized as ambivalent or uncertain about DBS.

It is possible that PD patients may not be fully aware of the full range of benefits that DBS therapy can provide; and instead believe that DBS is helpful only for a single symptom such as tremor (25). Another possible explanation for the uncertainty around DBS efficacy is that patient goals of surgical therapy may not align with what DBS can reasonably offer. Up to 25% of patients may still express dissatisfaction with DBS despite improvement of motor control (26), as patients tend to desire treatments that improve non-motor symptoms as well (27). Research has found that non-motor symptoms, which are not directly improved by DBS, are negatively associated with patient quality of life (28). Thus, the inability of DBS to address these symptoms may contribute to a patient ambiguity or ambivalence about the effectiveness of DBS. This preference may explain why some patients do not consider DBS to be “effective,” as their expectations may extend beyond the control of motor symptoms. Furthermore, patient goals may shift after DBS implantation—particularly when it comes to the importance of improvements in non-motor symptoms and behavioral goals (29).

### Timing of DBS Surgery

In the DBS-aware cohort, 55% of patients reported that they would wait at least 3 years before considering DBS; and over 60% of our DBS-aware cohort considered the treatment as a last resort therapy reserved only for advanced PD. This sentiment is consistent with prior studies (17, 19), with one in particular noting that patients qualifying for EARLYSTIM did not necessarily view themselves as ill enough to undergo DBS. Yet, the results of the EARLYSTIM study have suggested that DBS may be considered once motor complications lead to significant disability despite optimal medical management (30, 31). Interestingly, the correlation between medication failure and consideration of DBS was not clearly apparent in our cohort, as perceived efficacy of their current medical treatment was not associated with patients' willingness to undergo DBS. The persistent view of DBS as a last-resort therapy is particularly notable given recent findings suggesting that DBS performed within 4 years of diagnosis may modify disease progression (32–34).

### The Decision Burden: Balancing Implantation Benefits With Concerns About DBS Invasiveness, Reversibility, and Impacts on Normality

A stigma of “brain surgery” certainly exists. This stigma has been suggested in a study of epilepsy patients, in whom approximately half of patients were *unwilling* to undergo a hypothetical surgery even if it was deemed curative and without risk (14). We did not identify this same degree of hesitancy toward *hypothetical, risk-free curative* PD surgery in our cohort, suggesting that the stigma suggested in the epilepsy cohort does not generalize to Parkinson's patients. The fear of being awake for DBS surgery may represent a deterrent patients from pursuing surgical evaluation. Almost half of our cohort expressed this fear. Discussing the option of DBS implantation under general anesthesia, which has been shown to be as efficacious as its awake counterpart (35–40), may reduce this apprehension and thus help reduce the aforementioned under-utilization of DBS.

As must be done with all surgical interventions, DBS candidates must weigh the finite therapeutic benefits of DBS against the risk of complications. Fear of complications is often the primary contributing factor toward reluctance (13). Our observation that over twice as many patients in our cohort would agree to participate in medical trials of novel drugs to treat PD vs. novel surgeries may reflect increased concern regarding adverse events from brain surgery (14). That being said, it remains unclear if the risks associated with DBS surgery are significantly greater than with medical management. On the one hand, the rate of levodopa-associated dyskinesia is estimated to be 30–40% within 5 years according to recent literature (41). On the other hand, surgical complication rates of DBS for PD are estimated to be between 1 and 5% (42) and estimates of adverse effects possibly attributable to DBS stimulation (e.g., impaired speech or gait, depression, cognitive disturbances) have been estimated to be ~23% (43). Patients are tasked with imagining a life of living with device-based treatment and continuous neurostimulation. The experience of adapting to a possible new baseline of cognitive and motor functioning related to stimulation is a reality unique to DBS (44). While patients generally believe that the therapeutic benefits outweigh these impacts (9), this unique dimension of the decision burden may serve as a further barrier to patients considering DBS therapy. In our cohort, we also found an correlation between negative experiences with prior surgeries and a greater concern for alterations in mood and emotional well-being with DBS.

While surgery described “minimally invasive” or “reversible” was a factor in our cohort's consideration of an intervention, DBS may not resonate as either because it is a permanently implanted device. Almost twice as many PD patients would agree to a *reversible* PD surgery vs. *irreversible* PD surgery, but only 18.3% of our DBS-aware cohort agreed that the procedure was “reversible.” There are mixed perceptions about what is meant by a hypothetical return to pre-implantation baseline should patients undergo electrode removal (45). Because we did not specify in the survey what we meant by “reversible;” patients may have interpreted this question either as *implantation* reversibility or *stimulation* reversibility. From an implantation perspective, DBS causes distinct inflammatory changes and eventual glial scar formation (46, 47); however, longitudinal studies in animal models have demonstrated that these changes are largely confined to where electrodes were implanted (48). With regards to stimulation, plasticity induced by DBS stimulation may lead to retained clinical benefit (49). Given the possibility that stimulation induces permanent changes in neuronal structure or function, viewing DBS as truly *reversible* may be a mischaracterization even if a system can be turned off or removed. While patient knowledge of specific effects of stimulation may vary, it is possible given our data that many patients think that continuous neural stimulation is never truly reversible, even if it can theoretically be turned off. At the same time, in contrast to ablative procedures, such as radiofrequency or focused ultrasound lesioning, DBS is inherently more reversible because a permanent lesion is not created in the brain.

### Costs and Level of Disability as Determinants of Impressions of DBS

Over half of our DBS-aware cohort reported concerns about either insurance coverage or cost. Currently, DBS is covered by most insurance carriers including Medicare; however, factors such as race, sex, and neighborhood socio-economic status have been found to predict disparities in treatment among the insured (50). Studies have found implantation for both early and advanced PD to be cost-effective (with an estimated 1.7 QALYs added over best medical therapy) despite the upfront and maintenance costs (7, 51). Implanted individuals may also see reductions in the costs related to their medications (52). As such, when discussing costs, providers may benefit from discussing DBS implantation as a cost-effective treatment to improve quality of life. Further studies would be required to understand the true impact of this gap in understanding on the consideration of DBS.

### Limitations

The multiple-choice format of our questions allowed us to survey a high-volume of patients; but restricts the diversity of participant responses. The multiple-choice format did not include an “I do not know” option, and thus, we recognize that some patients may have answered “neutral” to indicate this response. In addition, we note that patients interpreted our questions subjectively, and the lack of free-response options constrains the patients' interpretations. It is important to note that this study was designed to allow for patient interpretation in order to better understand how terms commonly used to describe surgical procedures would affect their opinion of the intervention. Future areas of investigation can focus on asking patients about shared common experiences with DBS perceptions and allow for open responses to help provide further clarification.

While our results represent patients at US, urban, movement disorders clinics, from an urban American tertiary care clinic, we recognize that these findings may not be generalizable to other settings (e.g., international, rural areas). In addition, only a small minority of patients had not heard about DBS, and thus their perspectives may not be as well-represented in our study.

Cognitive impairment was an exclusion criteria; however, a complete mental status evaluation with scores (i.e., mini mental state examination) was not performed for each subject. Based on the neurologist assessment during the visit, patients who were dependent on a care-giver to respond to survey or with severe dementia were not offered to participate in the study. Therefore, there is a potential bias of patients with borderline cognitive decline who were not identified during clinical assessment.

Finally, we note that the patients recruited in our study are widely varied in disease duration, clinical staging, and therapeutic regimen. These factors all have the potential to impact a patient's quality of life and thus their willingness to accept DBS. While we attempted to elucidate how these factors may affect patient impressions *via* our analysis, we note that studies with more homogenous cohorts are necessary to connect impressions of DBS to clinical scales of PD.

## Conclusions

The decision to implant a device to modulate the brain is a complex, multifactorial decision that is ultimately driven by the individual and the information obtained from a variety of sources including physicians, the internet, other patients, family, friends, and patient support groups. Providers should have frank discussions with patients about their knowledge of DBS in order to assess their impressions and understanding of its effectiveness for PD symptoms as well as their goals for surgical therapy. Our data demonstrate that patients voiced greater overall concerns regarding procedural efficacy, invasiveness, cost, and lack of reversibility than concerns about threats to personality, mood and well-being, impacts on activities of daily living, and changes to appearance. Addressing barriers and correcting misperceptions about cost may help facilitate open discussions undergoing DBS.

While this study highlights certain impressions and gaps in understanding, future studies will be required to establish true causality and to allow us to better understand the impact of these initial findings on DBS hesitancy and underutilization.

## Data Availability Statement

The original contributions presented in the study are included in the article/Supplementary Material, further inquiries can be directed to the corresponding author/s.

## Ethics Statement

The studies involving human participants were reviewed and approved by Thomas Jefferson University Institutional Review Board. The patients/participants provided their written informed consent to participate in this study.

## Author Contributions

SD: research project (design, organization, and data collection) and manuscript (preparation and review). CM and LV: research project (data collection), data analysis (qualitative analysis and quantitative analysis), and manuscript (review). SR: research project (data collection) and manuscript (preparation and review). JB, SK, MR, T-WL, JR, and DK: research project (data collection) and manuscript (review). MB: research project (design) and manuscript (review). CW: research project (design, organization, and data collection), manuscript (preparation and review), and manuscript (preparation and review). All authors contributed to the article and approved the submitted version.

## Conflict of Interest

T-WL: DBS study entitled Progress—Investigator. CW: Abbott Corp—Consultant; Boston Scientific—Consultant; Medtronic, Inc.—Consultant; Neuropace, Inc.—Consultant; Nevro Corp.—Consultant; Micro Systems Engineering, Inc.—Advisory Board. The remaining authors declare that the research was conducted in the absence of any commercial or financial relationships that could be construed as a potential conflict of interest.

## Supplementary Material

Supplementary Figure 1 Survey distributed to recruited patients.
