# Supplementary material for: Capturing Initial Understanding and Impressions of Surgical Therapy for Parkinson's Disease
Source: Front Neurol. 2021 Mar 4;12:605959. doi: 10.3389/fneur.2021.605959 (PMC7970030; doi:10.3389/fneur.2021.605959)
Supplement: Supplementary Figure 1 — Survey distributed to recruited patients. [file Data_Sheet_1.pdf]

BEFORE YOU BEGIN:

Please answer all questions to the best of your ability. The person administering this survey is not permitted to explain information about the contents of this survey, but he/she may help you record your answers and/or determine which questions to answer. Your responses are 100% confidential and will in no way impact the care you receive.

Thank you for your participation.

SECTION 1

1.1 Have you ever had surgery before?

YES **NO**

(1.2) If YES, please describe the most recent surgery you received:

____

____

____

(1.3) If YES, how would you describe your most recent surgical experience?

___ **negative**
___ **somewhat negative**
___ **neutral**
___ **somewhat positive**
___ **positive**

1.4 Hypothetically, would you consider having brain surgery if it were guaranteed to cure your Parkinson's Disease and if the procedure was completely risk-free?

YES **NO**

SECTION 2

In the following section, please circle the appropriate number indicating your level of agreement with the following statements regarding brain surgery for Parkinson's Disease.

		Strongly Disagree				*Strongly Agree*
2.1	My current treatment(s) including medication, exercise, rehabilitation, and/or natural therapies for Parkinson's disease is/are effective at managing mysymptoms.	**1**	**2**	**3**	**4**	**5**
2.2	Parkinson's disease progresses and gets worse over time.	**1**	**2**	**3**	**4**	**5**
2.3	I would consider having ***reversible*** brain surgery to treat my Parkinson's Disease, if it was the best treatment option available.	**1**	**2**	**3**	**4**	**5**
2.4	I would consider having ***irreversible*** brain surgery to treat my Parkinson's Disease, if it was the best treatment option available.	**1**	**2**	**3**	**4**	**5**
2.5	I would consider having ***open*** brain surgery to treat my Parkinson's Disease, if it was the best treatment option available.	**1**	**2**	**3**	**4**	**5**
2.6	I would consider having ***minimally-invasive*** brain surgery to treat my Parkinson's Disease, if it was the best treatment option available.	**1**	**2**	**3**	**4**	**5**
2.7	I would consider having ***incisionless*** brain surgery to treat my Parkinson's Disease, if it was the best treatment option available.	**1**	**2**	**3**	**4**	**5**

		Strongly Disagree				***Strongly Agree***
2.8	There will be a cure for Parkinson's Disease in less than 10 years	**1**	**2**	**3**	**4**	**5**
2.9	I would be willing to participate in a clinical trial for a new **surgically implanted device** designed to treat Parkinson's Disease.	**1**	**2**	**3**	**4**	**5**
2.10	I would be willing to participate in a clinical trial for a new **surgical procedure** designed to treat Parkinson's Disease.	**1**	**2**	**3**	**4**	**5**
2.11	I would be willing to participate in a clinical trial for new **drug** designed to treat Parkinson's Disease.	**1**	**2**	**3**	**4**	**5**

SECTION 3

3.1 Are you familiar with Deep Brain Stimulation?

YES **NO**

- If answer is **NO**, **please stop here.**
- If answer is **YES, please complete the rest of the survey.**

3.2 How did you firsthear about DBS? [check all that apply]

____ **Internet (e.g. Google search, advertisement, webinar)**
____ **Friend**
____ **Physician**
____ **Family Member**
____ **Live Educational Event (support group, seminar)**
____ **Other (please specify in the space provided):**

: ____

SECTION 4

In the following section, please circle the appropriate number indicating your level of agreement with the following statements.

		Strongly Disagree				*Strongly Agree*
4.1	Deep Brain Stimulation is an **effective** treatment for Parkinson's Disease.	**1**	**2**	**3**	**4**	**5**
4.2	Deep Brain Stimulation is an **invasive** procedure.	**1**	**2**	**3**	**4**	**5**
4.3	Deep Brain Stimulation is **reversible.**	**1**	**2**	**3**	**4**	**5**
4.4	I am concerned that Deep Brain Stimulation would alter my **appearance.**	**1**	**2**	**3**	**4**	**5**
4.5	I am concerned that Deep Brain Stimulation would alter my **personality.**	**1**	**2**	**3**	**4**	**5**
4.6	I am concerned that Deep Brain Stimulation would alter my **mood and emotional well-being**.	**1**	**2**	**3**	**4**	**5**
4.7	I am concerned that Deep Brain Stimulation would negatively impact my ability to perform **daily tasks such as driving**	**1**	**2**	**3**	**4**	**5**
4.8	I am concerned that Deep Brain Stimulation is very **expensive**.	**1**	**2**	**3**	**4**	**5**
4.9	I am concerned that Deep Brain Stimulation is **not** covered by **insurance**.	**1**	**2**	**3**	**4**	**5**

		Strongly Disagree				*Strongly Agree*
4.10	I am concerned that Deep Brain Stimulation is a **new and unproven** technology.	**1**	**2**	**3**	**4**	**5**
4.11	I believe that Deep Brain Stimulation should only be considered as a **last resort** for patients with advanced Parkinson's Disease.	**1**	**2**	**3**	**4**	**5**
4.12	I am afraid of being awake during the Deep Brain Stimulation Procedure.	**1**	**2**	**3**	**4**	**5**

4.13 If your PD symptoms worsened, would youconsider undergoing DBS in: (check the earliest option you would consider.)

_____ **1 year or less**
_____ **Between 1 to 3year(s)**
_____ **Between 3 to 5years**
_____ **Between 5 to 10 years**
_____ **10+ years**
_____ **I would *never* consider undergoing DBS.**
